# Internal exposure levels of polycyclic aromatic hydrocarbons in children and adolescents: a systematic review and meta-analysis

**DOI:** 10.1186/s12199-019-0805-9

**Published:** 2019-07-27

**Authors:** Xin Huang, Xu Deng, Wenyan Li, Shudan Liu, Yiwen Chen, Bo Yang, Qin Liu

**Affiliations:** School of Public Health and Management, Research Center for Medicine and Social Development, Collaborative Innovation Center of Social Risks Governance in Health, Chongqing Medical University, No. 1 Yixueyuan Road Yuzhong District, Chongqing, 400016 China

**Keywords:** Polycyclic aromatic hydrocarbons, Children, Biological markers, Systematic review, Meta-analysis

## Abstract

Polycyclic aromatic hydrocarbons (PAHs) are widely found in the environment, and comparing to adults, children are more vulnerable to PAHs exposure. Urinary metabolites of PAHs are used as preferred biomarkers to estimate the PAHs exposure. Systematic review on the internal exposure level of children and adolescents is rare. We aimed to calculate the internal exposure levels of PAHs in children and adolescents and compare the levels of PAHs internal exposure in various children groups. We searched PubMed, OVID, Web of Science, EBSCO, ACS, and four Chinese databases, and all studies examining the urinary concentrations of PAHs in children and adolescent were identified. The total exposure level of 11 PAHs metabolites were pooled. Standard mean difference (SMD) and 95% confidence intervals (CIs) of PAHs urinary concentration were calculated and pooled by RevMan5.3 to compare the exposure levels of different children groups. We found that 1-OHPyr, 2-OHNap, 2-OHFlu, 3-OHPhe, and 4-OHPhe were five PAHs metabolites most commonly studied in existing studies in children, and their total exposure levels were 0.38 ± 0.98, 2.32 ± 4.83, 0.81 ± 1.54, 0.09 ± 0.14, 0.03 ± 0.10 μmol/mol creatinine, respectively. The meta-analysis showed that the levels of 1-OHPyr were higher in higher environmental exposure group (SMD = 0.21, 95% CI = 0.03~0.40), ETS exposure group (SMD = 0.31, 95% CI = 0.08~0.54), and 6~11 years group (SMD = 0.16, 95% CI = 0.09~0.24); the level of 2-OHNap (SMD = 0.27, 95% CI = 0.01~0.53) was higher in higher environmental exposure group; however, the levels of 3-OHPhe (SMD = − 0.34, 95% CI = − 0.57~− 0.12) and 4-OHPhe (SMD = − 0.48, 95% CI = − 0.69~− 0.28) were higher in lower environmental exposure group. The levels of 1-OHPyr (SMD = − 0.01, 95% CI = − 0.11~0.10) and 2-OHNap (SMD = 0.01, 95% CI = − 0.20~0.22) were not statistically different between boys and girls. In conclusions, we found that the internal diversity of PAHs existed in children and adolescents, and the level of 1-OHPyr in children and adolescents was in higher status compared with non-occupational people who do not smoke.

**Electronic supplementary material:**

The online version of this article (10.1186/s12199-019-0805-9) contains supplementary material, which is available to authorized users.

## Introduction

Polycyclic aromatic hydrocarbons (PAHs) are a group of over 100 different organic pollutants, which are widely found in the environment [1]. The major sources of PAHs are formed during the incomplete burning of coal, oil and gas, garbage, or other organic substances like tobacco or charbroiled meat [2]. Due to the numerous PAHs exposure sources, humans can be exposed to PAHs through multiple routes, including breathing polluted air, environmental tobacco smoke (ETS), dietary PAHs intake, and dermal absorption through soil, air, or particulate deposited on skin [3–5].

The exposure of PAHs in human has raised public health concerns. The United States Environmental Protection Agency (USEPA) has designated 16 PAH compounds as priority pollutants [3, 6]. Among PAHs, benzo[a]pyrene (BaP) has been classified as a probable human and animal carcinogen by the International Agency for Research on Cancer (IARC) [7, 8]. The exposure of PAHs has been linked to the onset of diabetes mellitus [9], metabolic syndrome [10], and cardiovascular conditions [11]. Several studies reported that genotoxicity [12], oxidative stress [13], asthma [14], and neurodevelopment [15] were related to the exposure of PAHs. Besides, it has been found that PAHs exposure may be association with Cytochrome P450s (CYPs) induction and bioactivation, thereby leading to carcinogenesis [16]. Furthermore, existing studies showed that children are more vulnerable to PAHs exposure. Huang et al. reported that urinary 1-hydroxypyrene (1-OHPyr) concentration in children (6–11 years old) was approximately 30% higher compared to that in adults under the same conditions, indicating that children seem more susceptible to PAHs and have higher potential health risks [50].

Compared to monitoring of the external environment (e.g., measurement of chemicals in air, water, or soil), human biomonitoring reflects internal exposure in the human through different routes of exposure [17]. After entering the human body and biotransformation, PAHs are excreted in the form of hydroxylated metabolites in the urine or stool [18]. Biomarkers can provide an integrated reflection for exposure through inhalation, food and dermal uptake, and takes into account variation in absorption, metabolism, and elimination by the body. Therefore, the urinary metabolites of these compounds are used as preferred biomarkers to estimate the PAHs exposure [19].

To date, a number of studies have detected the urinary metabolites concentration of PAHs; however, the biomarkers and characteristics were diverse. Several studies conducted a review on the concentration of urinary metabolites and biomarkers of PAHs. A comprehensive review on occupational exposure to PAHs by Bouchard et al. [20] revealed that urinary 1-OHPyr increased in course of a working day. Hansen et al. [21] reviewed 132 studies addressing the use of 1-OHPyr as a biomarker of both occupational and environmental exposure to PAH. Of these, 25 studies addressed environmental exposure, and only nine included children. The existing reviews on PAHs metabolites concentration are focused more on adults, but comprehensive review on the internal exposure level of children and adolescence is rare. The purpose of this study was to estimate the total non-occupational internal exposure level in children using different hydroxylated metabolites and to compare the levels of PAHs internal exposure in various children groups. Furthermore, a reference for future research and evaluation on PAHs biomarkers in children will be provided from the estimated total internal exposure level.

## Methods

### Selection criteria

The inclusion criteria included (1) studies that reported the urinary concentrations of PAHs metabolites; (2) participants: children and adolescents whose age under 20 years; and (3) outcome: concentration of PAHs hydroxylated metabolites (the preferred indicators were mean and standard deviation, median and range were included after calculating by formula). We excluded studies if (1) the studies were reviews, editorials, meeting abstracts, or commentaries; and (2) the small sample size studies detected concentration of PAHs to verify the feasibility of detection method. We did not have language restrictions.

### Search strategy

The databases including PubMed (1978 to January 2017), OVID (1946 to January 2017), Web of Science (1970 to January 2017), EBSCO (1976 to January 2017), ACS(1879 to January 2017), CNKI (1979 to January 2017), WANFANG DATA (1987 to January 2017), CBM (1978 to January 2017), and CQVIP (1989 to January 2017) were searched using both the MeSH terms and free terms “Polycyclic Aromatic Hydrocarbons” or “PAHs” or “hydroxypyrene” or “hydroxyfluorene” or “hydroxyphenanthrene” or “hydroxynaphalene” or “hydroxyfluoranthrene” or “hydroxybenzo[c]phenanthrene” or “hydroxychrysene” or “hydroxybenz[a]anthracene,” in combination with “urinary.” We modified the search strategy when searching in different databases. No language restrictions or restrictions on publication type were applied. All the retrieved literatures were entered into reference-managing software (EndNote, version X6, Thomson Scientific, Stamford, CT, USA) for duplicate check.

### Data screening and extraction

Two reviewers independently screened all the retrieved literature by titles and abstracts. The potential eligible studies were then screened again by full texts. The pre-designed criteria mentioned above were used to guide the entire process of screening. Subsequently, the following data were extracted from all the included studies using a pre-designed extraction form by two reviewers: (1) general information, including authors, publication year, country; (2) study design and methodological quality; (3) participants characteristics and sample size; (4) sample collection season; (5) analyte detection and adjusted methods; and (6) outcome measures, including type of metabolites, outcome indicators, and concentrations of metabolites. Disagreements during screening and data extraction were resolved by discussion or consultation with the third reviewer to reach a consensus.

### Risk of bias assessment

Two reviewers independently assessed the methodology quality of included studies using the checklist recommended by Agency for Healthcare Research and Quality (AHRQ) [22]. Every item should be answered by “Yes,” “No,” or “Unclear.” Disagreements were resolved by discussion or consultation with the third reviewer to reach a consensus.

### Recalculation of urinary PAHs

The given concentrations were recalculated where the concentrations were given in the unit of ng/g creatinine, ng/mg creatinine, and nmol/mol creatinine. For the recalculation molecular weight of hydroxypyrene (218.25), hydroxynaphthalene (144.17), hydroxyfluorene (182.22), hydroxyphenanthrene (194.23), and creatinine (113.12) were used. Outcome reported with median and ranges or median and interquartile range were converted to means and standard deviations according to the formula for approximately estimating [23, 24].

### Statistical analysis

**Fig. 1:**
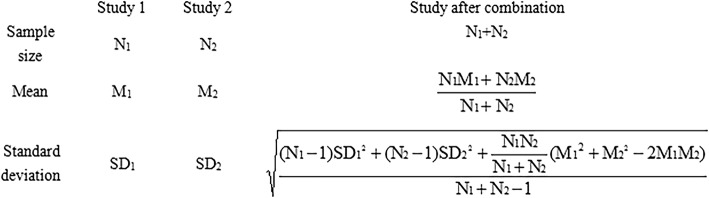
Formula of combination

A statistical formula (Fig. 1) was used to synthesize means and standard deviations to get a total level of PAHs metabolites. The standard mean difference (SMD) and 95% confidence interval (95% CI) of concentrations of PAHs metabolites were used to conduct meta-analyses using the software of Review Manager Software (Version5.3, Cochrane Collaboration, London, UK) for comparison between different children groups. The statistical heterogeneity of the included studies was assessed by *χ*^2^ test and *I*^2^ index. A random effects model was used when heterogeneity was found to be significant (*I*^2^ > 50% or *P* < 0.05); otherwise, the fixed effects model was used. Subgroup analysis was conducted according to different study period, countries, ages, sample collection seasons, and approximately estimating or not. Sensitivity analyses were conducted using the leave-one-out approach for all the outcomes. To examine the potential publication bias, we used the funnel plot firstly. When larger and smaller studies were non-symmetrically distributed, visual inspection of the funnel plot offered an indication of publication bias. The presence of publication bias was further tested using Begg’s test and Egger’s test by STATA 12.0 software (StataCorp LP, College Station, TX). A *P* ≤ 0.05 was considered to be statistically significant.

## Results

### Screening results

**Fig. 2:**
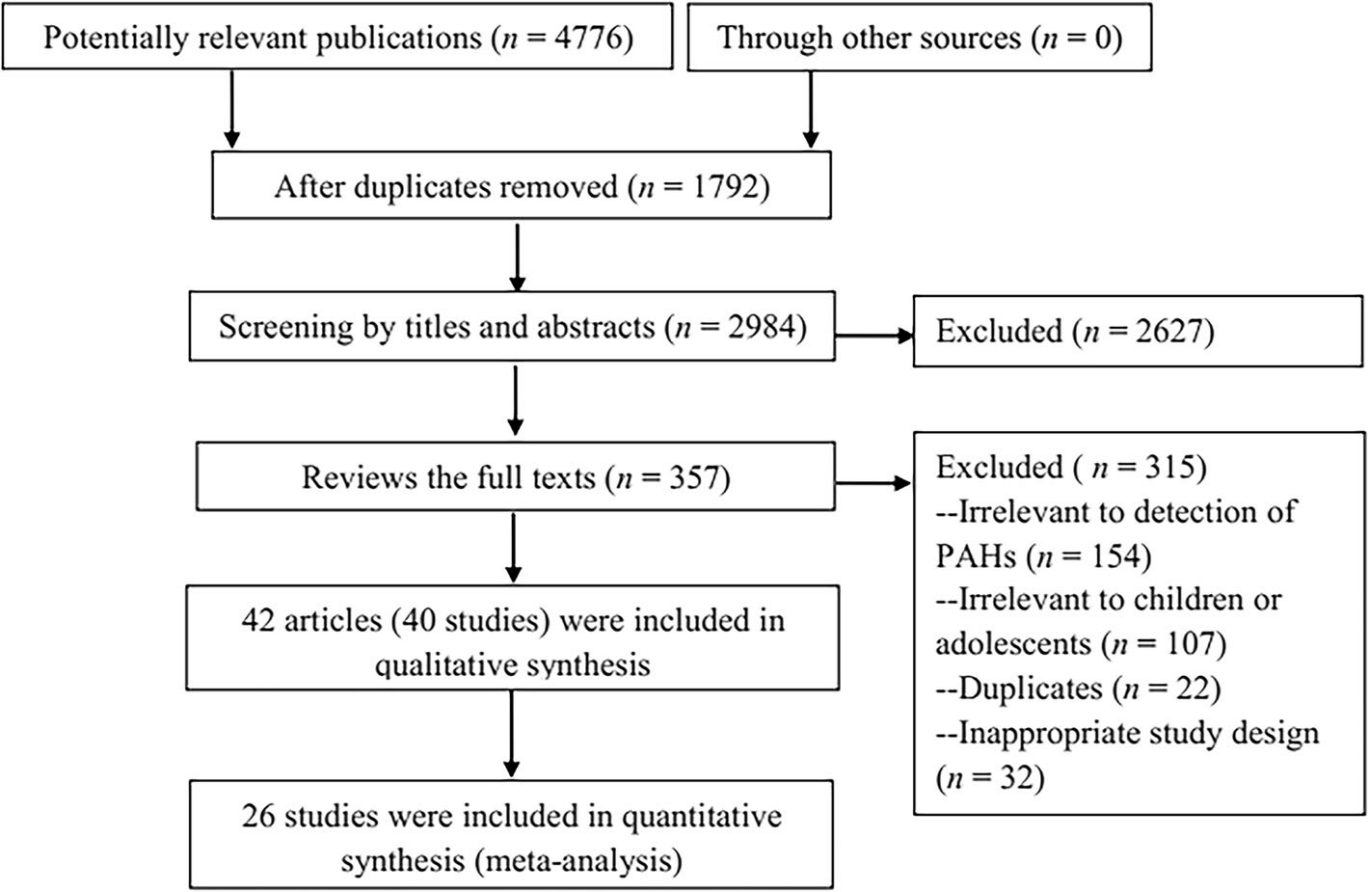
Flow diagram of literature search

The process of study selection is shown in Fig. 2 with an adapted PRISMA (Preferred reporting items for systematic reviews and meta-analyses) flow diagram [25]. A total of 4776 records were identified after searching the literature. After duplicate checking, 2984 studies were excluded after the initial screening of titles and abstracts. Forty studies described in 42 articles [26–67] involving 12697 subjects were included in the final review after a strict screening process based on eligibility criteria, of which, 26 studies [26–53, 66] were included in the meta-analysis. The other 315 studies were excluded because they did not report the concentration of PAHs, or were not relevant to children and adolescent, or duplicate publication or studies were reviews, meeting abstracts, or commentaries.

### Characteristics of included studies

Table 1 presents the main characteristics of included studies. The earliest study began in 1993. Seventeen [27, 28, 30–32, 38, 41–43, 47, 50, 52, 53, 56, 59, 60, 64, 67] of 40 studies were performed in developed countries or regions, and 23 [26, 29, 33–37, 39, 40, 44–46, 48, 49, 51, 54, 55, 57, 58, 61–63, 65, 66] were conducted in developing countries. A total of 12697 children were enrolled in this review, of which age range were 0~20 years. Six studies [39, 46, 48, 61, 63, 64] did not mentioned the age of subjects, but they reported that the subjects were all the elementary school students. Six studies [45, 48, 54, 55, 59, 62] detected the level of PAHs metabolites only in boys, the remaining [26–44, 46, 47, 49–53, 56–58, 60, 61, 63–67] included both girls and boys. Five out of 40 studies [30–32, 50, 52, 56] determined the PAHs metabolites by gas chromatography (GC), the remaining [26–29, 33–49, 51, 53–55, 57–67] used the high-performance liquid chromatography (HPLC) method. The numbers of metabolites were from one to 11, and all the studies reported concentration of 1-OHPyr. Of the 42 articles based on the 40 studies, 29 [26, 28, 29, 32–34, 36, 38–44, 46, 48, 49, 53–64] reported the level of PAHs by mean and standard deviation, five [30, 31, 50–52] reported by mean and its 95% CI, three [27, 45, 67] reported by mean and range, three [47, 65, 66] reported by median and range, and two [35, 37] reported by median and interquartile range. Thirty-seven of 40 studies reported the PAHs metabolites concentrations adjusted by creatinine, one study in two papers [30, 31] reported the concentrations adjusted by urine specific gravity (SG), and two studies [51, 64] reported the unadjusted concentrations. Sixteen studies compared the urine PAHs metabolites levels between children living in higher PAHs exposure environment and lower exposure environment. The higher living environment included the urban areas [27, 32, 38, 41, 45], industrial city or areas [40, 46, 48, 49], commercial areas [39], and schools or communities close to main roads [44], steel plant [43], steel mill and coking facility [33], power plant [42], coke oven plant [47], or oil refinery [37]. Oppositely, the rural areas [27, 32, 38, 41, 45, 47], tourist city [40], agricultural or residential areas [46, 48, 49], and schools or communities far away from the industry plants [33, 37, 42–44] were regarded as the lower living environment.

**Table 1:** General characteristics of included studies

Study	Country/Areas	Study period	Sample size (Boys/Girls)	Age range	Sample collection season	Analyte detection	Adjusted methods	Metabolites	Outcome indicators
Chen 2015 [26]	Mongolia	2011–2012	320 (166/154)	11–15 years	Spring, autumn, winter	HPLC-FD	Creatinine	1-OHPyr	Mean ± SD
Hansen 2005 [27]	Denmark	1994–1995	204 (108/96)	7–8 years	Winter	HPLC-FD	Creatinine	1-OHPyr	Mean (range)
Kang 2002 [28]	Korea	1997	137 (78/59)	11–14 years	Summer	HPLC-FD	Creatinine	1-OHPyr, 2-OHNap	Mean ± SD
Mielzynska 2006 [29]	Poland	1998–1999	74 (47/27)	5–14 years	Winter	HPLC-FD	Creatinine	1-OHPyr	Mean ± SD
Miller 2010 [30]	USA	1998–2006	222 (119/103)	5 years	Irregular	GC-MS/MS	SG	1-OHPyr, 1-OHNap, 2-OHNap, 2-OHFlu, 3-OHFlu, 9-OHFlu, 1-OHPhe, 2-OHPhe, 3-OHPhe, 4-OHPhe	Mean (95% CI)
Jung 2015 [31]				9 years					
Morgan 2015 [32]	USA	2000–2001	126 (63/63)	2– years	Spring, summer, autumn	GC/MS	Creatinine	1-OHPyr	Mean ± SD
Mucha 2006 [33]	Ukraine	1998	90 (43/47)	3 years	Spring	HPLC	Creatinine	1-OHPyr	Mean ± SD
Ochoa -Martinez 2016 [34]	Mexico	2012	135(NA)	6–12 years	Not mentioned	HPLC-FD	Creatinine	1-OHPyr	Mean ± SD
Yue 2010(1) [35]	China	2007	80 (47/33)	4–6 years	Winter	HPLC-FD	Creatinine	1-OHPyr	Median (interquartile range)
Yue 2011 [36]								2-OHFlu, 2-OHPhe, 3-OHPhe, 4-OHPhe, 9-OHPhe	Mean ± SD
Alghamdi 2015 [37]	Saudi Arabian	2013	170 (NA)	11 years	Spring	HPLC	Creatinine	1-OHPyr, 3-OHFlu, 1-OHPhe, 2-OHPhe, 3-OHPhe, 4-OHPhe	Median (interquartile range)
Cirillo 2006 [38]	Italian	2004	30 (15/15)	7–9 years	Winter	HPLC-UV	Creatinine	1-OHPyr	Mean ± SD
Duan 2003 [39]	China	2002	40 (NA)	Not mentioned	Winter	HPLC	Creatinine	1-OHPyr	Mean ± SD
Fan 2007 [40]	China	2005	108 (72/36)	16–18 years	Spring, summer	HPLC-MS/MS	Creatinine	1-OHPyr, 1-OHNap, 2-OHNap, 2-OHFlu, 2-OHPhe, 3-OHPhe, 4-OHPhe, 9-OHPhe	Mean ± SD
Freire 2009 [41]	Spain	2005–2006	174 (NA)	4 years	Irregular	HPLC	Creatinine	1-OHPyr	Mean ± SD
Hu 2011 [42]	Taiwan/China	2009	369 (198/171)	1–13 years	Autumn, winter	HPLC	Creatinine	1-OHPyr	Mean ± SD
Lee 2007 [43]	Korea	2004	1012 (475/537)	7–15 years	Spring	HPLC	Creatinine	1-OHPyr	Mean ± SD
Ruchirawat 2007 [44]	Thailand	2006	176 (NA)	9–13 years	Not mentioned	HPLC	Creatinine	1-OHPyr	Mean ± SD
Shahsavani 2016 [45]	Iran	2015	120 (120/0)	9–12 years	Spring	HPLC	Creatinine	1-OHPyr	Mean (range)
Su 2015 [46]	China	2014	164 (NA)	Not mentioned	Autumn	HPLC-MS	Creatinine	1-OHPyr, 1-OHNap, 2-OHNap, 2-OHFlu, 3-OHFlu, 1-OHPhe, 2-OHPhe, 3-OHPhe, 4-OHPhe, 9-OHPhe	Mean ± SD
Wilhelm 2007 [47]	Germany	2000	215 (NA)	4–9 years	Spring	HPLC	Creatinine	1-OHPyr	Median (range)
Yang 1997 [48]	China	1994	88 (88/0)	Not mentioned	Summer, winter	HPLC	Creatinine	1-OHPyr	Mean ± SD
Yu 2012 [49]	China	2010–2011	185 (NA)	9–12 years	Winter	HPLC	Creatinine	1-OHPyr	Mean ± SD
Huang 2006 [50]	USA	1999–2000	1003 (NA)	6–19 years	Not mentioned	GC/IDHRMS	Creatinine	1-OHPyr	Mean (95%CI)
Huang 2014 [51]	China	2009–2010	2015 (NA)	6–20 years	Not mentioned	HPLC	Unadjusted	1-OHPyrr, 1-OHNap, 2-OHNap, 3-OHPhe	Mean (95%CI)
Li 2008 [52]	USA	2001–2002	1122 (NA)	6–19 years	Not mentioned	GC/IDHRMS	Creatinine	1-OHPyr, 1-OHNap, 2-OHNap, 2-OHFlu, 3-OHFlu, 9-OHFlu, 1-OHPhe, 2-OHPhe, 3-OHPhe, 4-OHPhe, 9-OHPhe	Mean (95% CI)
Heudorf 2001 [53]	Germany	1998	718 (NA)	0–20 years	Spring	HPLC-FD	Creatinine	1-OHPyr, 1-OHPhe, 2-OHPhe, 3-OHPhe, 4-OHPhe	Mean ± SD
Yue 2010(2) [54]	China	2006	15 (15/0)	14–18 years	Winter	HPLC-FD	Creatinine	1-OHPyr, 2-OHNap, 2-OHFlu, 2-OHPhe, 3-OHPhe, 4-OHPhe, 9-OHPhe	Mean ± SD
Yue 2009 [55]	China	2005	41 (41/0)	16–18 years	Summer	HPLC-FD	Creatinine	1-OHPyr, 2-OHNap, 2-OHFlu, 2-OHPhe, 3-OHPhe, 4-OHPhe, 9-OHPhe	Mean ± SD
Farzan 2016 [56]	USA	2003–2008	660(NA)	12–19 years	Not mentioned	GC/IDHRMS	Creatinine	1-OHPyr, 1-OHNap, 2-OHNap, 2-OHFlu, 3-OHFlu, 9-OHFlu, 1-OHPhe, 2-OHPhe, 3-OHPhe, 4-OHPhe	Mean ± SD
Perez -Maldonado 2014 [57]	Mexico	2008–2009	226 (112/114)	6–12 years	Not mentioned	HPLC	Creatinine	1-OHPyr	Mean ± SD
Martinez -Salinas 2010 [58]	Mexico	2010	258 (NA)	3–13 years	Not mentioned	HPLC	Creatinine	1-OHPyr	Mean ± SD
Cavanagh 2007 [59]	New Zealand	2004	89 (89/0)	12–18 years	Autumn, winter	HPLC	Creatinine	1-OHPyr	Mean ± SD
Wijnen 1996 [60]	The Netherlands	1992	644 (NA)	1–6 years	Summer	HPLC-FD	Creatinine	1-OHPyr	Mean ± SD
Ma 1996 [61]	China	1992–1994	574 (NA)	Not mentioned	Summer, winter	HPLC	Creatinine	1-OHPyr	Mean ± SD
Zhao 1995 [62]	China	1991–1992	310 (310/0)	6–15 years	Spring, autumn, winter	HPLC	Creatinine	1-OHPyr	Mean ± SD
Ma 1994 [63]	China	1992	145 (NA)	Not mentioned	Autumn, winter	HPLC	Creatinine	1-OHPyr	Mean ± SD
Kanoh 1993 [64]	Japan	1988–1989	139 (NA)	Not mentioned	Summer, winter	HPLC	Unadjusted	1-OHPyr	Mean ± SD
Siwińska 1998 [65]	Poland	1996	30 (18/12)	8 years	Not mentioned	HPLC	Creatinine	1-OHPyr	Median (range)
Siwińska 1999 [66]	Poland	1996	412(NA)	7–8 years	Not mentioned	HPLC	Creatinine	1-OHPyr	Median (range)
Fiala 2001 [67]	The Czech Republic	1997–1998	57(NA)	3–6 years	Summer, winter	HPLC-FD	Creatinine	1-OHPyr	Mean (range)

*OHPyr* hydroxypyrene, *OHNap* hydroxynaphalene, *OHFlu* hydroxyfluorene, *OHPhe* hydroxyphenanthrene, *HPLC* high-performance liquid chromatography, *FD* fluorescence detection, *UV* ultraviolet, *MS* mass spectrometry, *GC* gas chromatography, *IDHRMS* isotope dilution high-resolution mass spectrometry, *SG* specific gravity, *SD* standard deviation, *NA* not available

### Risk of bias in included studies

The quality assessment of cross-sectional studies was shown in Table 2. Of the 11 items in quality assessment, all the studies reported the data sources, inclusion and exclusion criteria for exposed and unexposed subjects, study period, and all the subjects were consecutive; it is unclear in all studies that whether or not the evaluators of subjective components were masked to other aspects of the status of the participants; only two studies [51, 64] did not adjust the levels of PAHs; five studies [30, 31, 33, 42, 43, 45] described the reasons for sample exclusions from analysis; 15 studies [34, 40, 44, 46–49, 56–58, 60, 63, 65–67] did not describe how to control confounding factors; only two studies [43, 45] explained how to deal with the data below the limit of detection; 11 studies [27, 33, 35–37, 42, 43, 45, 47, 50, 51, 60] described the completeness of data collection; and four studies [26, 30, 31, 43, 48] had follow-ups and reported the percentage of patients obtained in follow-ups.

**Table 2:** AHRQ for assessing the methodology quality of included studies

Study	(1)	(2)	(3)	(4)	(5)	(6)	(7)	(8)	(9)	(10)	(11)
Chen 2015 [26]	Y	Y	Y	Y	U	Y	N	Y	N	N	Y
Hansen 2005 [27]	Y	Y	Y	Y	U	Y	N	Y	N	Y	N
Kang 2002 [28]	Y	Y	Y	Y	U	Y	N	Y	N	N	N
Mielzynska 2006 [29]	Y	Y	Y	Y	U	Y	N	Y	N	N	N
Miller 2010 [30]	Y	Y	Y	Y	U	Y	Y	Y	N	Y	Y
Jung 2015 [31]											
Morgan 2015 [32]	Y	Y	Y	Y	U	Y	N	Y	N	N	N
Mucha 2006 [33]	Y	Y	Y	Y	U	Y	Y	Y	N	Y	N
Ochoa-Martinez 2016 [34]	Y	Y	Y	Y	U	Y	N	N	N	N	N
Yue 2010(1) [35]	Y	Y	Y	Y	U	Y	N	Y	N	Y	N
Yue 2011 [36]											
Alghamdi 2015 [37]	Y	Y	Y	Y	U	Y	N	Y	N	N	N
Cirillo 2006 [38]	Y	Y	Y	Y	U	Y	N	Y	N	N	N
Duan 2003 [39]	Y	Y	Y	Y	U	Y	N	Y	N	N	N
Fan 2007 [40]	Y	Y	Y	Y	U	Y	N	N	N	N	N
Freire 2009 [41]	Y	Y	Y	Y	U	Y	N	Y	N	N	N
Hu 2011 [42]	Y	Y	Y	Y	U	Y	Y	Y	N	Y	N
Lee 2007 [43]	Y	Y	Y	Y	U	Y	Y	Y	Y	Y	Y
Ruchirawat 2007 [44]	Y	Y	Y	Y	U	Y	N	N	N	N	N
Shahsavani 2016 [45]	Y	Y	Y	Y	U	Y	Y	Y	Y	Y	N
Su 2015 [46]	Y	Y	Y	Y	U	Y	N	N	N	N	N
Wilhelm 2007 [47]	Y	Y	Y	Y	U	Y	N	N	N	Y	N
Yang 1997 [48]	Y	Y	Y	Y	U	Y	N	N	N	N	Y
Yu 2012 [49]	Y	Y	Y	Y	U	Y	N	N	N	N	N
Huang 2006 [50]	Y	Y	Y	Y	U	Y	N	Y	N	Y	N
Huang 2014 [51]	Y	Y	Y	Y	U	N	N	Y	N	Y	N
Li 2008 [52]	Y	Y	Y	Y	U	Y	N	Y	N	N	N
Heudorf 2001 [53]	Y	Y	Y	Y	U	Y	N	Y	N	N	N
Yue 2010(2) [54]	Y	Y	Y	Y	U	Y	N	Y	N	N	N
Yue 2009 [55]	Y	Y	Y	Y	U	Y	N	Y	N	N	N
Farzan 2016 [56]	Y	Y	Y	Y	U	Y	N	N	N	N	N
Perez-Maldonado 2014 [57]	Y	Y	Y	Y	U	Y	N	N	N	N	N
Martinez-Salinas 2010 [58]	Y	Y	Y	Y	U	Y	N	N	N	N	N
Cavanagh 2007 [59]	Y	Y	Y	Y	U	Y	N	Y	N	N	N
Wijnen 1996 [60]	Y	Y	Y	Y	U	Y	N	N	N	Y	N
Ma 1996 [61]	Y	Y	Y	Y	U	Y	N	Y	N	N	N
Zhao 1995 [62]	Y	Y	Y	Y	U	Y	N	Y	N	N	N
Ma 1994 [63]	Y	Y	Y	Y	U	Y	N	N	N	N	N
Kanoh 1993 [64]	Y	Y	Y	Y	U	N	N	Y	N	N	N
Siwińska 1998 [65]	Y	Y	Y	Y	U	Y	N	N	N	N	N
Siwińska 1999 [66]	Y	Y	Y	Y	U	Y	N	N	N	N	N
Fiala 2001 [67]	Y	Y	Y	Y	U	Y	N	N	N	N	N

(1) Define the source of information (survey, record review); (2) list inclusion and exclusion criteria for exposed and unexposed subjects (cases and controls) or refer to previous publications; (3) indicate time period used for identifying patients; (4) indicate whether or not subjects were consecutive if not population-based; (5) indicate if evaluators of subjective components of study were masked to other aspects of the status of the participants; (6) describe any assessments undertaken for quality assurance purposes (e.g., test/retest of primary outcome measurements); (7) explain any patient exclusions from analysis; (8) describe how confounding was assessed and/or controlled; (9) if applicable, explain how missing data were handled in the analysis; (10) summarize patient response rates and completeness of data collection; (11) clarify what follow-up, if any, was expected and the percentage of patients for which incomplete data or follow-up was obtained. *Y* yes, *N* no, *U* unclear

### Total exposure levels of PAHs

In this study, the concentration of 11 PAHs metabolites in urine of children and adolescents were pooled based on the 37 studies [26–29, 32–50, 52–63, 65–67] reporting the concentrations adjusted by creatinine respectively. 1-OHPyr, 2-OHNap, 2-OHFlu, 3-OHPhe, and 4-OHPhe were five PAHs metabolites most commonly used in existing studies, and their total exposure levels were 0.38 ± 0.98, 2.32 ± 4.83, 0.81 ± 1.54, 0.09 ± 0.14 and 0.03 ± 0.10 μmol/mol creatinine, respectively. Among three age groups, the concentration of 9-OHPhe (1.90 ± 1.20) in children under six were higher than those in other age groups; the concentration of 1-OHPyr (0.45 ± 0.75) in children aged 6 to 11 years was higher than that in other groups. Between the age groups of 6 to 11 years and 12 to 20 years, the concentration of 1-OHNap (1.95 ± 2.56), 2-OHNap (2.37 ± 5.34), 3-OHFlu (0.09 ± 0.22), and 9-OHFlu (0.15 ± 0.36) in adolescent aged 12 to 20 years were higher than those in six to 11 years group; the concentration of 2-OHFlu (1.48 ± 0.20), 1-OHPhe (0.09 ± 0.17), 2-OHPhe (0.07 ± 0.19), 3-OHPhe (0.14 ± 0.20), and 4-OHPhe (0.04 ± 0.15) in children aged 6 to 11 years were higher than those in 12 to 20 years age groups. Details are shown in Table 3.

**Table 3:** Concentration of 11 PAHs metabolites in children and adolescents

Metabolites	No. of studies	Concentrations in different age groups (Mean ± SD, μmol/mol Cre.)			
		< 6 years	6–11 years	12–20 years	Total
1-OHPyr	37	0.24 ± 0.35	0.45 ± 0.75	0.18 ± .0.52	0.38 ± 0.98
1-OHNap	4	–	1.78 ± 3.07	1.95 ± 2.56	1.90 ± 2.78
2-OHNap	7	–	2.18 ± 2.67	2.37 ± 5.34	2.32 ± 4.83
2-OHFlu	6	–	1.48 ± 0.20	0.38 ± 1.38	0.81 ± 1.54
3-OHFlu	2	–	0.07 ± 0.19	0.09 ± 0.22	0.08 ± 0.21
9-OHFlu	2	–	0.11 ± 0.20	0.15 ± 0.36	0.14 ± 0.33
1-OHPhe	3	–	0.09 ± 0.17	0.07 ± 0.11	0.08 ± 0.13
2-OHPhe	4	–	0.07 ± 0.19	0.06 ± 0.07	0.07 ± 0.12
3-OHPhe	5	–	0.14 ± 0.20	0.07 ± 0.10	0.09 ± 0.14
4-OHPhe	8	–	0.04 ± 0.15	0.02 ± 0.05	0.03 ± 0.10
9-OHPhe	4	1.90 ± 1.20	0.02 ± 0.02	0.16 ± 0.45	0.29 ± 0.76

The concentration of 2-OHNap, 1-OHNap, 2-OHFlu, 3-OHFlu, 9-OHFlu, 1-OHPhe, 2-OHPhe, 3-OHPhe, and 4-OHPhe in children aged under 6 years were not pooled due to the lack of data.

### Comparison of PAHs metabolites levels in various children groups

#### 1-OHPyr

##### Living environment and 1-OHPyr levels

**Fig. 3:**
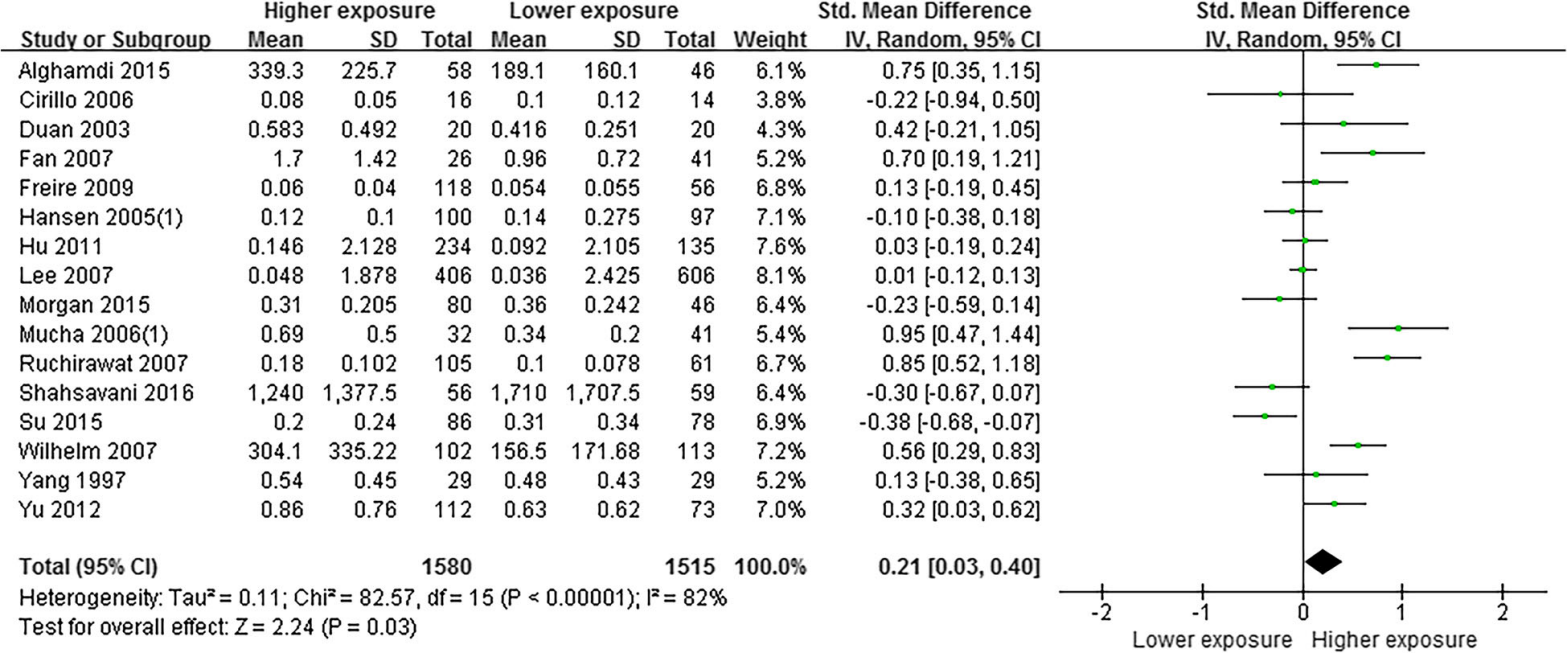
Meta-analysis on differences of level of 1-OHPyr in children and adolescents with different living condition

Meta-analysis based on 16 studies [27, 32, 33, 37–49] indicated that the level of 1-OHPyr was higher in children living with higher environmental exposure than that in children living with lower exposure (SMD = 0.21, 95% CI = 0.03~0.40) (Fig. 3). A random effects model was adopted (*P* < 0.05, *I*^2^ = 82%). Subgroup analyses by countries, ages, gender, type of data, study period, and sample collection seasons are shown in Table 4. Statistical differences in levels of 1-OHPyr between higher exposure group and lower exposure group were found in subgroups of developing countries (SMD = 0.37, 95% CI = 0.03~0.72), both genders (SMD = 0.25, 95% CI = 0.04~0.46), and study period in 2001–2010 (SMD = 0.27, 95% CI = 0.04~0.50).

**Table 4:** Subgroup analysis on differences of level of 1-OHPyr in children and adolescents with different living condition

Character	Subgroup	No. studies	SMD	95% CI		Heterogeneity			Begg’s test		Egger’s test	
				Lower	Upper	*χ*^2^	*P*	*I*^2^ (%)	*z*	*P*	*t*	*P*
Overall		16	0.21	0.03	0.40	82.57	< 0.01	82	0.77	0.44	1.32	0.21
Countries (Additional file 1: Figure S1)	Developed	7	0.06	− 0.12	0.23	18.05	< 0.01	67	− 0.45	0.65	0.05	0.98
	Developing	9	0.37	0.03	0.72	53.81	< 0.01	85	0.21	0.84	3.28	0.41
Age (Additional file 1: Figure S2)	≤ 6 years	3	0.26	− 0.33	0.86	14.51	< 0.01	86	0.00	1.00	1.15	0.46
	7–18 years	11	0.19	− 0.05	0.43	56.93	< 0.01	82	0.47	0.64	0.98	0.35
Gender (Additional file 1: Figure S3)	Boys only	3	0.03	− 0.40	0.45	4.42	0.11	55	1.57	0.12	5.58	0.02
	Both genders	13	0.25	0.04	0.46	76.37	< 0.01	84	1.10	0.27	2.35	0.17
Data (Additional file 1: Figure S4)	Original	12	0.21	0.00	0.42	55.76	< 0.01	80	0.69	0.49	1.70	0.22
	Estimating	4	0.22	− 0.24	0.69	25.27	< 0.01	88	− 0.68	0.49	0.58	0.96
Study period (Additional file 1: Figure S5)	1994–2000	5	0.25	− 0.16	0.66	25.49	< 0.01	84	0.24	0.81	1.63	0.77
	2001–2010	8	0.27	0.04	0.50	31.19	< 0.01	78	0.87	0.39	2.17	0.16
	2011–2015	3	0.02	− 0.66	0.69	21.24	< 0.01	91	1.04	0.30	19.77	0.40
Sample collection season (Additional file 1: Figure S6)	Spring	5	0.37	− 0.04	0.77	38.48	< 0.01	90	1.47	0.14	3.61	0.25
	Winter	5	0.12	− 0.11	0.35	5.83	0.21	31	0.00	1.00	0.18	0.93
	Unclear	6	0.17	− 0.19	0.53	37.93	< 0.01	87	0.94	0.35	3.28	0.53

##### Environmental tobacco smoke and 1-OHPyr levels

**Fig. 4:**
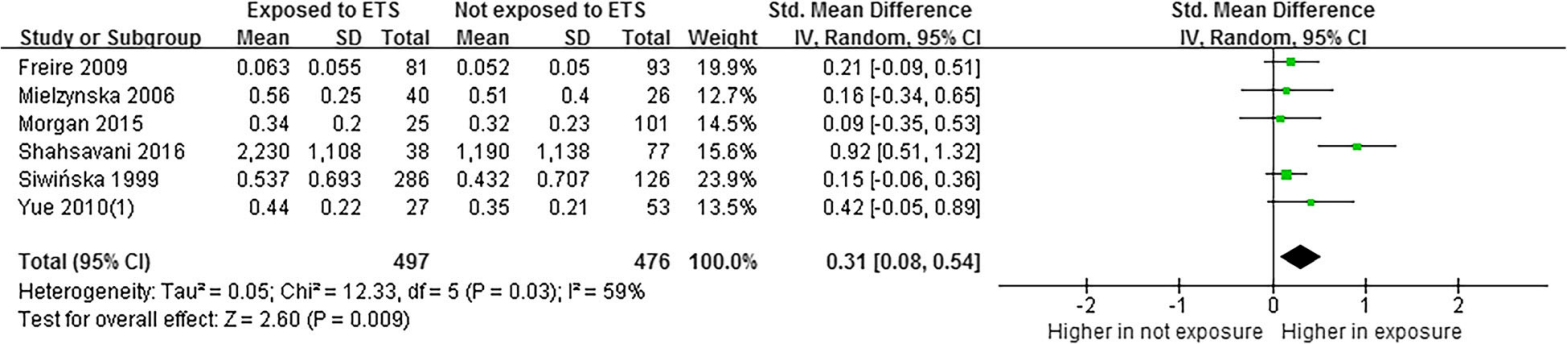
Meta-analysis on differences of level of 1-OHPyr in children and adolescents with ETS

Meta-analysis based on six studies [29, 32, 35, 41, 45, 66] indicated that the level of 1-OHPyr was higher in children exposed to ETS than that in children not exposed to ETS (SMD = 0.31, 95% CI = 0.08~0.54) (Fig. 4). A random effects model was adopted (*P* < 0.05, *I*^2^ = 59%). The pooled results were not changed in each individual sensitivity analysis by the leaving one out approach.

##### Gender and 1-OHPyr levels

**Fig. 5:**
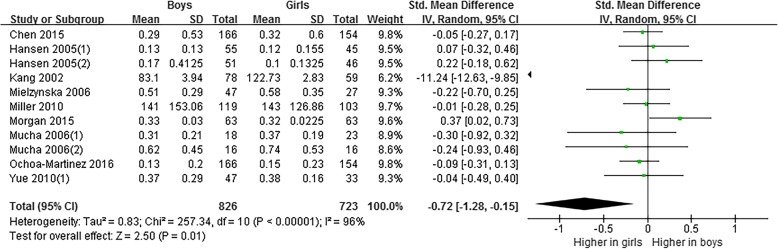
Meta-analysis on differences of level of 1-OHPyr in children and adolescents by gender

**Fig. 6:**
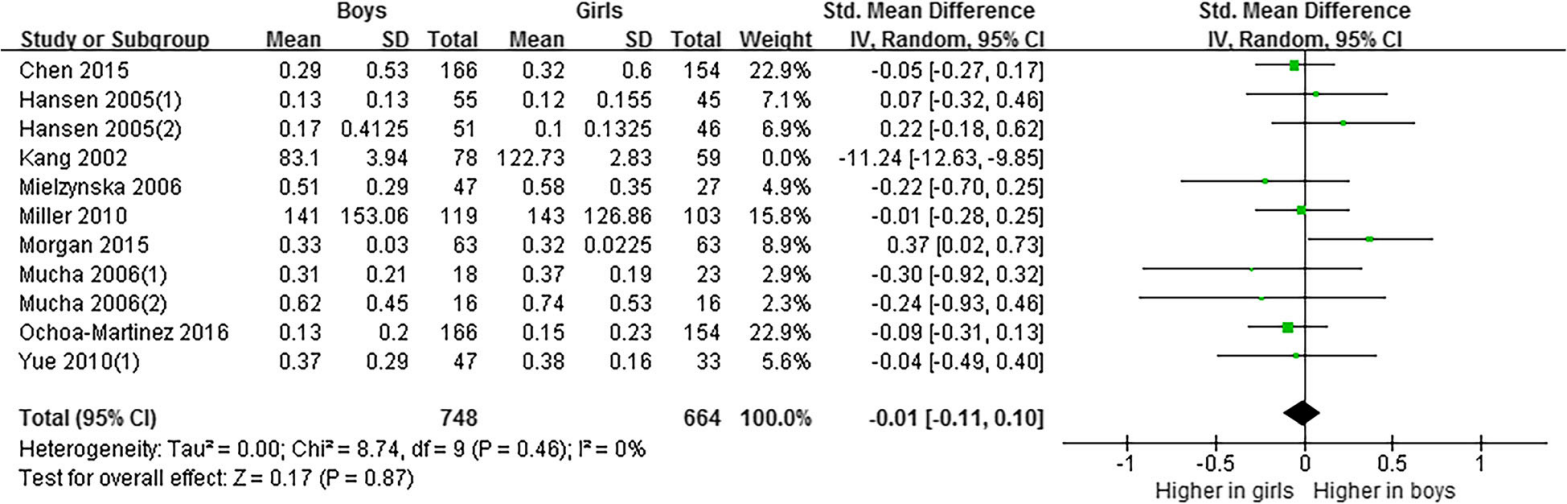
Sensitivity analysis of meta-analysis on differences of level of 1-OHPyr in children and adolescents by gender

Meta-analysis based on 1549 subjects [26–35] indicated that the level of 1-OHPyr in girls were higher than that in boys (SMD = − 0.72, 95% CI = − 1.28~− 0.15) (Fig. 5). A random effects model was adopted (*P* < 0.05, *I*^2^ = 96%). However, after removing Kang 2002 [28] in sensitivity analysis, the pooled data changed to no statistical difference between genders (SMD = − 0.01, 95% CI = − 0.11~0.10), and the heterogeneity reduced to 0% (Fig. 6).

##### Age and 1-OHPyr levels

**Fig. 7:**
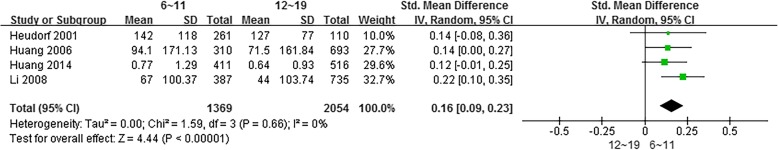
Meta-analysis on differences of levels of 1-OHPyr in children and adolescents in different age groups

Four studies [50–53] pooled the data of 1-OHPyr in different age group, showing that the concentration of 1-OHPyr in the 6~11 years group was higher than that in 12~19 years group (SMD = 0.16, 95% CI = 0.09~0.23), (Fig. 7). The fixed effects model was adopted (*P* > 0.05, *I*^2^ = 0%).

#### 2-OHNap

##### Living environment and 2-OHNap levels

**Fig. 8:**
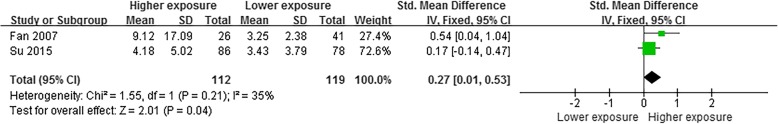
Meta-analysis on differences of level of 2-OHNap in children and adolescents with different living condition

Two studies [40, 46] reported the concentration of 2-OHNap. The pooled data showed that the concentration of 2-OHNap detected in children living with higher PAHs exposure was higher than that in children with lower exposure (SMD = 0.27, 95% CI = 0.01~0.53); the fixed effects model was adopted (*P* > 0.05, *I*^2^ = 35%), (Fig. 8).

##### Gender and 2-OHNap levels

**Fig. 9:**
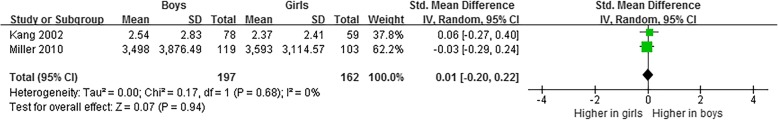
Meta-analysis on differences of level of 2-OHNap in children and adolescents by gender

Two studies [28, 30] pooled the data of concentration of 2-OHNap, showing that there were no statistical differences in the level of 2-OHNap between boys and girls (SMD = 0.01, 95% CI = − 0.20~0.22); the fixed effects model was adopted (*P* > 0.05, *I*^2^ = 0%), (Fig. 9).

#### Other metabolites

Two studies [40, 46] pooled the data of 1-OHNap and 2-OHPhe, and three studies [37, 40, 46] pooled the data of 3-OHPhe and 4-OHPhe. The pooled data showed no significant differences in urinary concentration of 1-OHNap, 2-OHPhe and 3-OHPhe between the groups living with higher/lower environmental exposure (Table 5), while the concentration of 4-OHPhe detected in children living with lower exposure was higher than that in children with higher exposure (SMD = − 0.48, 95% CI = − 0.69~− 0.28, Additional file 1: Figure S11). When the data of Fan 2007 [40] was removed in sensitivity analysis of 3-OHPhe, the pooled result altered (SMD = − 0.34, 95%CI = − 0.57~− 0.12), and the heterogeneity reduced to 0% (Additional file 1: Figure S10).

**Table 5:** Urinary concentration of four PAH metabolites between living with higher environmental exposure group and living with lower environmental exposure group

Metabolites	No. of studies	Model	SMD (95%CI)	*p*	Heterogeneity		
					*χ*^2^	*p*	*I*^2^
1-OHNap (Additional file 1: Figure S7)	2	Fixed effects	0.35 [− 1.28,1.99]	0.67	1.59	0.21	37%
2-OHPhe (Additional file 1: Figure S8)	2	Random effects	0.02 [− 0.11, 0.16]	0.75	5.30	0.02	81%
3-OHPhe (Additional file 1: Figure S9)	3	Random effects	− 0.16 [− 0.55,0.23]	0.42	6.46	0.04	69%
	2^a^	Fixed effects	− 0.34 [− 0.57, − 0.12]	0.003	0.29	0.59	0%
4-OHPhe (Additional file 1: Figure S11)	3	Fixed effects	− 0.48 [− 0.69, − 0.28]	< 0.01	1.45	0.48	0%

^a^Sensitivity analysis of meta-analysis on differences of levels of 3-OHPhe in children and adolescents with different living conditions (Additional file 1: Figure S10)

## Discussion

PAHs are widely diffused in the environment, and there is a high risk for human to expose to it. Knowing the exposure level of PAHs and comparing the levels in various groups can provide basic data for the environment pollution control, and can also contribute to the further study on association of PAHs with human health. A total of 12697 children were enrolled in this study, covering 19 countries or areas of five continents, detecting 11 kinds of PAHs urinary metabolites. To the best of our knowledge, this is the first systematic review and meta-analysis to estimate the total level of non-occupational internal exposure of PAHs and to compare exposure levels of PAHs metabolites in various groups of children and adolescents.

The present study pooled the total concentration of 11 PAHs metabolites in children and adolescents based on 37 studies including 10321 children. 1-OHPyr is the most widely used biomarker of PAHs, and several [68–70] studies reported the occupational exposure limit (OEL) of 1-OHPyr. However, there is lack of the limit value for general population, nor for children. Jongeneeleen et al. [68] suggested that the reference value in non-occupational people is 0.24 μmol/mol creatinine and 0.76 μmol/mol creatinine for non-smokers and smokers, respectively; however, the pooled value of 1-OHPyr in children in this study was 0.38 μmol/mol creatinine, which is higher than the reference value. 1-OHNap and 2-OHNap were the highest levels of PAHs metabolites in the urine of children, with the pooled value of 1.90 and 2.32 μmol/mol creatinine, respectively. Except for 1-OHPyr, all the other ten PAHs biomarkers have no reference or limit value; the pooled concentration in this study could provide a reference for future research and evaluation on PAHs biomarkers in children. Compared to the general population, the levels of 1-OHPyr and 3-OHPhe in this study were higher than those in the USA [56] and lower than those in China [51]. Contrarily, the levels of 1-OHNap and 2-OHNap were higher than those in China [51] and lower than those in the USA [56].

This study found that level of 1-OHPyr in children living with higher environmental exposure was higher than that in children living with lower exposure. The level of 1-OHPyr in urine is influenced by many factors, including biological variability between subjects (e.g., age, gender, race, health status, nutritional status), cultural differences (e.g., environment, diet, smoking, alcohol, occupation), and variation in the laboratory techniques applied (e.g., sampling design, protocols, analytical precision of the measurement) [21]. Subgroup analysis was conducted to explore the potential confounding factors in this meta-analysis. We found that compared with children living in lower PAHs exposure area, children living with higher PAHs exposure had a higher level of 1-OHPyr in subgroup of developing countries and both genders. The results consist with a cross-sectional study [71] conducted in general population from seven Asian countries. Several studies [72–74] have shown that the concentration of 1-OHPyr was influence by smoking. In this study, we found that the level of 1-OHPyr also higher in children who was exposed to the ETS. The reason was most because of inducing effect of cigarette smoke on CPYs [75]. As for the gender, the pooled estimates showed that the level of 1-OHPyr has statistical differences between boys and girls with heterogeneity of 96%, which may not necessarily mean that the level of 1-OHPyr in girls was higher than that in boys. The inconsistency may be related to the study of Kang et al. [28]; children included in this study have sample bias. First, the study chose four schools which had different PAHs exposure levels in air. Second, the study only enrolled boys in a lower exposure school and enrolled girls in a higher exposure school. Thus, the level of 1-OHPyr in girls in this study was higher than boys. After removing Kang et al. [28] in meta-analysis, the pooled data altered, and the heterogeneity was reduced to 0%. Therefore, we suggest that the levels of 1-OHPyr were not statistically different between boys and girls. Moreover, the pooled data showed that the level of 1-OHPyr in children aged 6 to 11 years was higher than that in age group of 12~19 years. Behaviors that are common in childhood but are not observed in adults and the biological characteristics of young children may be reasons that lower age group has higher level of 1-OHPyr. On the one hand, young children prefer to play and crawl around on the floor and ground, increasing the risk of inhaling or dermally absorbing toxicants from particles, vapors present in carpets and soil [76]. On the other hand, previous studies [77, 78] have indicated that the metabolism of PAHs in children is different from that in adults, and children seem more susceptible to PAHs.

We found that the concentration of 2-OHNap detected in children living with higher environmental PAH exposure was higher than that in the lower exposure area. Li et al. [52] reported that concentration of 2-OHNap was well correlated with total PAH exposure, which indicated the higher level of PAHs in air, and the higher level in urine. There were no statistical differences in the levels of 2-OHNap between boys and girls. However, only two studies were included in the analysis, which may not reflect the real differences. Therefore, studies on 2-OHNap still need to be done to identify the potential differences between genders.

The meta-analysis only compared the concentration of other PAHs metabolites in children living with different environmental exposure. The pooled data showed that the levels of 3-OHPhe and 4-OHPhe detected in children living with lower environmental exposure were higher than those in the higher exposure area. Scinicariello et al. [79] noted that the main source of exposure of the larger PAHs (such as phenanthrene) is dietary, which could lead to the higher concentration of hydroxyphenanthrene found in lower exposure environment.

We conducted a comprehensive search to cover all the available studies measuring PAHs metabolites in children with non-occupational exposure to estimate the level of the PAHs internal exposure and to compare the levels of PAHs internal exposure in various children groups, the findings of which may provide a reference for studies in this area. However, several limitations still need to be considered when interpreting and generalizing the present results. First, some data had been recalculated before putting in the analysis which may influence the accuracy of the pooled estimates. The concentration of 2-OHNap, 1-OHNap, 2-OHFlu, 3-OHFlu, 9-OHFlu, 1-OHPhe, 2-OHPhe, 3-OHPhe, and 4-OHPhe in children aged under 6 years were not pooled due to the lack of data. Second, the level of PAHs in children may be abnormal distribution, and a mean value measures the central tendency, which could also lead to a bias in the estimating of total PAHs metabolites level and meta-analysis. Third, subgroup analyses were conducted by countries, gender, age, type of data, study period, and sample collection season in this study but the heterogeneity were not decrease obviously. In addition, heterogeneity that comes from ethnicity diversity, food intake differences, sample size differences, and health status could not be analyzed in the current meta-analysis, which also needs to be considered when interpreting the pooled results. Besides, we found that combustion of industrial sources and automobile exhaust were the main exposure sources of higher environmental exposure group in this study. However, the subgroup analysis grouping by kind of exposure sources were not conducted due to only one study [44] has tested the 1-OHPyr of children living near to main roads. Fourth, meta-analysis on differences of 1-OHPyr concentration in children and adolescents by age were conducted in children aged 6 to 11 years and 12 to 19 years. The cut-off point of 11 years may not represent a sharp limit, and the difference of levels of 1-OHPyr in children under 6 years group were not analyzed. Further, although majority of children of our included studies were recruited from schools, a small proportion of adolescents (12.11%) aged 12 to 20 years were sampled based on population registry, which may lead to including young people as “non-occupationally exposed” with the same likelihood of school children. Finally, except for 1-OHPyr, there were few studies with small sample size reported on other ten PAHs metabolites, thus the internal exposure levels in various children groups cannot be accurately analyzed.

## Conclusion

In summary, 1-OHPyr is the most widely used biomarker of PAHs. The pooled value of 1-OHPyr in children in this study was higher than the reported reference value of non-occupational people who do not smoke. The present meta-analysis suggests that the level of 1-OHPyr in children and adolescent were in relative high status, especially among the children at younger age. The internal diversity of PAHs exists in children and adolescents. Children and adolescents living with higher environmental exposure and exposed to ETS have higher concentration of 1-OHPyr. There were few studies reported on other PAHs metabolites such as 2-OHNap, 2-OHFlu, 3-OHPhe, and 4-OHPhe, thus more attention needs to be paid on those metabolites in future studies.

## Additional file

Additional file 1 **Figure S1.** Subgroup analysis by countries on differences of levels of 1-OHPyr in children and adolescents with different living conditions. **Figure S2.** Subgroup analysis by age groups on differences of levels of 1-OHPyr in children and adolescents with different living conditions. **Figure S3.** Subgroup analysis by gender on differences of levels of 1-OHPyr in children and adolescents with different living conditions. **Figure S4.** Subgroup analysis by type of data on differences of levels of 1-OHPyr in children and adolescents with different living conditions. **Figure S5.** Subgroup analysis by study period on differences of levels of 1-OHPyr in children and adolescents with different living conditions. **Figure S6.** Subgroup analysis by sample collection season on differences of levels of 1-OHPyr in children and adolescents with different living conditions. **Figure S7.** Meta-analysis on differences of levels of 1-OHNap in children and adolescents with different living conditions. **Figure S8.** Meta-analysis on differences of levels of 2-OHPhe in children and adolescents with different living conditions. **Figure S9.** Meta-analysis on differences of levels of 3-OHPhe in children and adolescents with different living conditions. **Figure S10.** Sensitivity analysis of meta-analysis on differences of levels of 3-OHPhe in children and adolescents with different living conditions. **Figure S11.** Meta-analysis on differences of levels of 4-OHPhe in children and adolescents with different living conditions. (DOCX 194 kb)

## Data Availability

Please contact author for data requests.

## Abbreviations

95% CI: 95% Confidence interval
AHRQ: Agency for Healthcare Research and Quality
BaP: Benzo[a]pyrene
CPYs: Cytochrome P450s
Cre.: Creatinine
ETS: Environmental tobacco smoke
FD: Fluorescence detection
GC: Gas chromatography
HPLC: High-performance liquid chromatography
IARC: International Agency for Research on Cancer
IDHRMS: Isotope dilution high resolution mass spectrometry
MS: Mass spectrometry
NA: Not available
OEL: Occupational exposure limit
OHFlu: Hydroxyfluorene
OHNap: Hydroxynaphalene
OHPhe: Hydroxyphenanthrene
OHPyr: Hydroxypyrene
PAHs: Polycyclic aromatic hydrocarbons
PRISMA: Preferred reporting items for systematic reviews and meta-analyses
SD: Standard deviation
SG: Specific gravity
SMD: Standard mean difference
USEPA: The United States Environmental Protection Agency
UV: Ultraviolet
