# Supplementary material for: Internal exposure levels of polycyclic aromatic hydrocarbons in children and adolescents: a systematic review and meta-analysis
Source: Environ Health Prev Med. 2019 Jul 27;24:50. doi: 10.1186/s12199-019-0805-9 (PMC6661086; doi:10.1186/s12199-019-0805-9)
Supplement: Supplementary file 1 — Figure S1. Subgroup analysis by countries on differences of levels of 1-OHPyr in children and adolescents with different living conditions. Figure S2. Subgroup analysis by age groups on differences of levels of 1-OHPyr in children and adolescents with different living conditions. Figure S3. Subgroup analysis by gender on differences of levels of 1-OHPyr in children and adolescents with different living conditions. Figure S4. Subgroup analysis by type of data on differences of levels of 1-OHPyr in children and adolescents with different living conditions. Figure S5. Subgroup analysis by study period on differences of levels of 1-OHPyr in children and adolescents with different living conditions. Figure S6. Subgroup analysis by sample collection season on differences of levels of 1-OHPyr in children and adolescents with different living conditions. Figure S7. Meta-analysis on differences of levels of 1-OHNap in children and adolescents with different living conditions. Figure S8. Meta-analysis on differences of levels of 2-OHPhe in children and adolescents with different living conditions. Figure S9. Meta-analysis on differences of levels of 3-OHPhe in children and adolescents with different living conditions. Figure S10. Sensitivity analysis of meta-analysis on differences of levels of 3-OHPhe in children and adolescents with different living conditions. Figure S11. Meta-analysis on differences of levels of 4-OHPhe in children and adolescents with different living conditions. (DOCX 194 kb) [file 12199_2019_805_MOESM1_ESM.docx]

**Supplementary Figures**


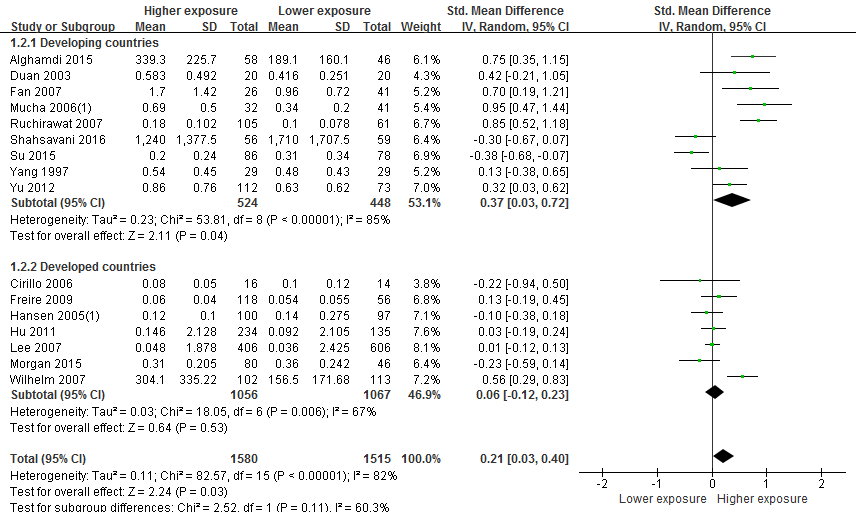


**Figure S1.** Subgroup analysis by countries on differences of levels of 1-OHPyr in children and adolescents with different living conditions


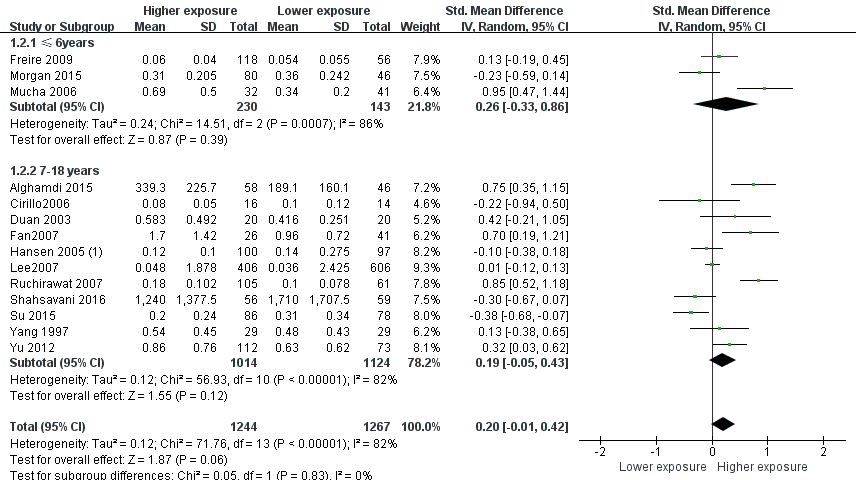


**Figure S2.** Subgroup analysis by age groups on differences of levels of 1-OHPyr in children and adolescents with different living conditions


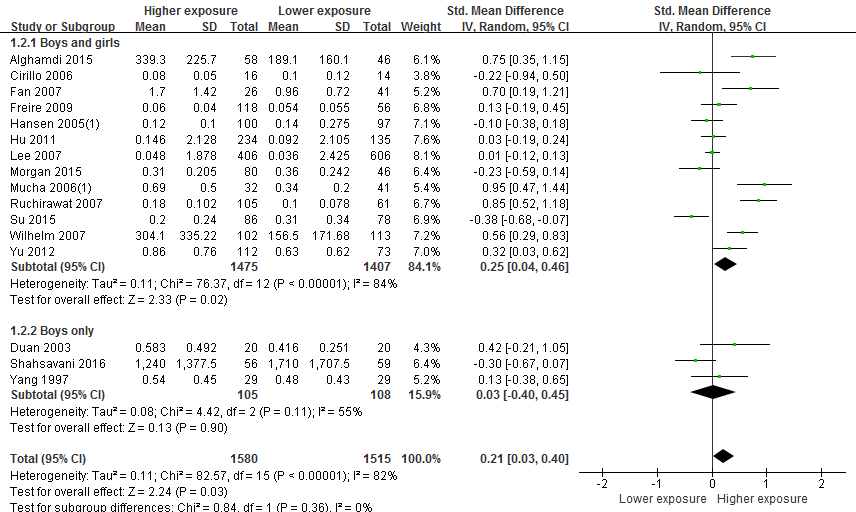


**Figure S3.** Subgroup analysis by gender on differences of levels of 1-OHPyr in children and adolescents with different living conditions


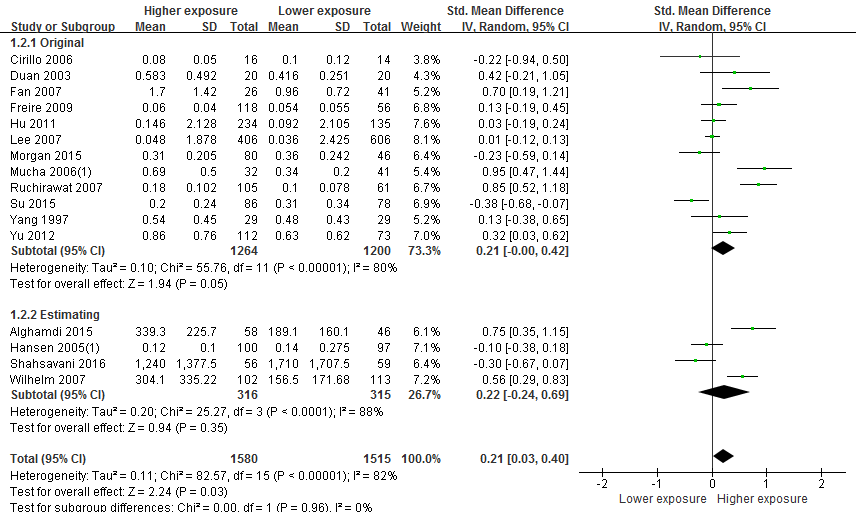


**Figure S4.** Subgroup analysis by type of data on differences of levels of 1-OHPyr in children and adolescents with different living conditions


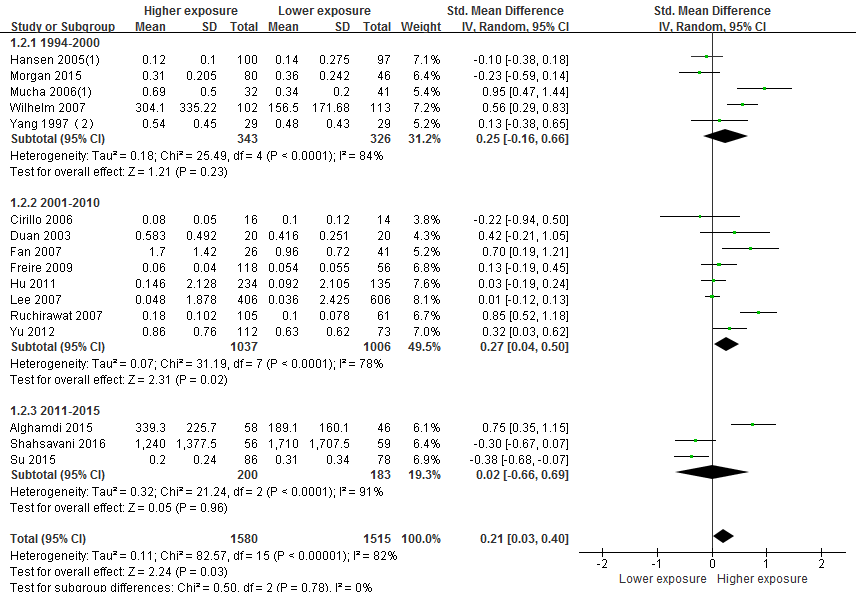


**Figure S5.** Subgroup analysis by study period on differences of levels of 1-OHPyr in children and adolescents with different living conditions


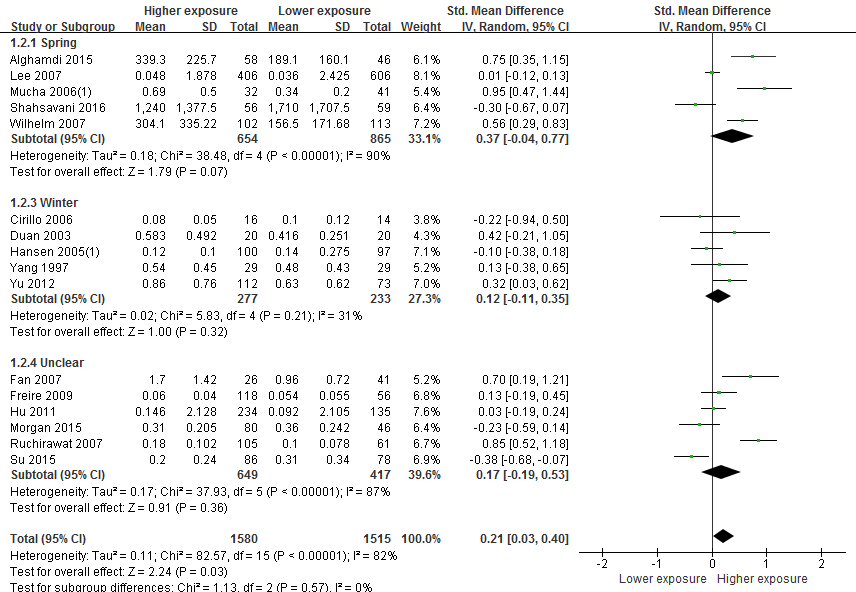


**Figure S6.** Subgroup analysis by sample collection season on differences of levels of 1-OHPyr in children and adolescents with different living conditions


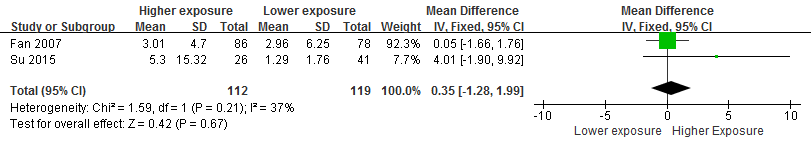


**Figure S7.** Meta-analysis on differences of levels of 1-OHNap in children and adolescents with different living conditions


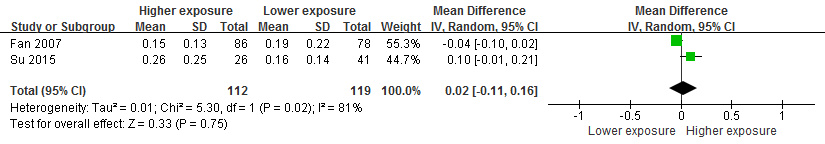


**Figure S8.** Meta-analysis on differences of levels of 2-OHPhe in children and adolescents with different living conditions


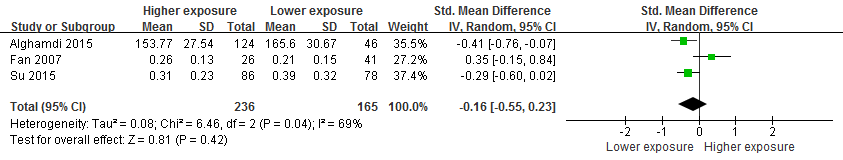


**Figure S9.** Meta-analysis on differences of levels of 3-OHPhe in children and adolescents with different living conditions


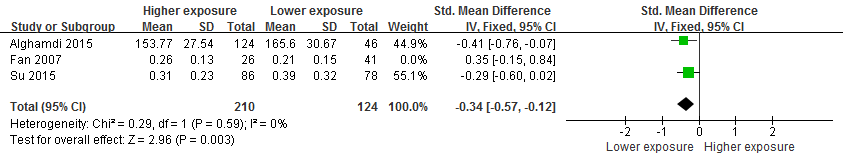


**Figure S10.** Sensitivity analysis of meta-analysis on differences of levels of 3-OHPhe in children and adolescents with different living conditions


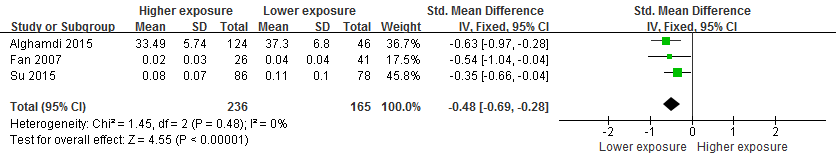


**Figure S11.** Meta-analysis on differences of levels of 4-OHPhe in children and adolescents with different living conditions
